# Changes in the incidence of early-onset breast cancer in Germany between 2010 and 2022

**DOI:** 10.1007/s10549-023-07048-1

**Published:** 2023-07-31

**Authors:** Niklas Gremke, Uwe Wagner, Matthias Kalder, Karel Kostev

**Affiliations:** 1 Department of Gynecology and Obstetrics, University Hospital Marburg, Philipps-University Marburg, Baldingerstraße, 35043 Marburg, Germany; 2 Institute of Molecular Oncology, Philipps-University Marburg, Hans-Meerwein-Straße 3, 35043 Marburg, Germany; 3 Epidemiology, IQVIA, Main Airport Center, Unterschweinstiege 2–14, 60549 Frankfurt, Germany

**Keywords:** Early-onset breast cancer, Breast cancer incidence, Age, Germany, Gynecologists, General practitioners

## Abstract

**Purpose:**

The aim of this study was to identify the mean age at which breast cancer (BC) was first diagnosed in 2010 or 2022, and to evaluate whether there were any changes in age groups at first BC diagnosis.

**Methods:**

This retrospective cross-sectional study included adult women (18 years or older) who were diagnosed with BC (ICD-10: C50) for the first time in 2010 or 2022 in office-based practices in Germany (in 300 general practices or 95 gynecological practices). We examined the mean age at diagnosis and the percentage of patients in three age groups (18–49, 50–65, and > 65) for both 2010 and 2022. The average age difference between 2010 and 2022 was analyzed using Wilcoxon rank tests, and the proportions of the three age groups were analyzed using chi-squared tests. These analyses were performed separately for patients in general and gynecological practices.

**Results:**

The mean age at which BC was initially diagnosed in 2022 was found to be significantly greater than that in 2010 for both general practices (66.9 years vs. 64.0 years *p* < 0.001) and gynecological practices (62.2 years vs. 60.3 years, *p* < 0.001). Early-onset BC decreased from 15.6 to 12.0% in general practices and from 23.2 to 18.2% in gynecological practices between 2010 and 2022. The proportion of new BC diagnoses in the age group 50–65 increased from 36.6 to 40.9% in gynecological practices, but did not increase in general practices.

**Conclusion:**

The study found that BC was diagnosed at an older age in 2022 than in 2010. In addition, the proportion of early-onset BC cases decreased, while the proportion of cases in the age group 50–65 increased in gynecological practices in Germany.

## Introduction

Breast Cancer (BC) is the most commonly diagnosed cancer worldwide and the leading cause of cancer death. There were an estimated 2.2 million new BC cases globally in 2020, including 191,105 new BC cases among 20–39 year old women [1, 2]. Importantly, BC incidence has increased continuously by 1.44% per year (95% CI 1.42, 1.47), with statistically significant increases in all age groups and in all regions worldwide except North America [3]. There is broad evidence that the rising incidence of cancer is linked to demographic shifts and could be partially attributed to the global increase in life expectancy. Particularly, in the US, the risk of developing cancer is nearly 10 times higher in persons aged 65 years than in persons under the age of 65 years, and it is estimated that 70% of all cancers will occur among adults aged ≥ 65 years by 2030 [4, 5]. In addition, changes in reproductive patterns and the use of postmenopausal hormone therapy may also contribute to the strong increase in BC incidence [6].

However, recent studies have also indicated a significant increase in the global burden of early-onset BC cancer and mortality among young adults, whereby early-onset BC is most often defined as the occurrence of a breast tumor before the age of 50 [7–10]. Notably, women who are diagnosed with BC at a younger age generally experience a more severe form of the disease than older women, resulting in a decreased likelihood of survival across all histological subtypes and stages despite receiving intensive treatment [11, 12]. Furthermore, there is growing evidence to suggest that younger BC patients are diagnosed at a more advanced tumor stage resulting in a less curable disease [13].

In particular, Heer et al. examined age-specific trends in BC incidence between 1971 and 2015 for Canada and found that BC incidence among women under the age of 40 has increased since 2000, while the incidence for those aged between 40 and 50 has remained stable [14]. For Europe, a plethora of study sets have calculated the annual percentage changes (APC) in BC incidence by age group and have yielded different results [13]. In a study focusing on patients from some seven European countries, Leclère and colleagues found a significant increase of + 2.0 in the APC value in the youngest age group (15–35) with BC, whereas Katalinic et al. reported a decrease of − 1.4 for women aged 20–49 years in Germany although the latter finding was not considered significant [15, 16].

In summary, several epidemiological studies from various countries have focused on incidence trends of early-onset BC and yielded inconsistent results when comparing younger and older BC patients, with a substantial degree of variation by geographical region. Aiming to generate further evidence on this topic, the objective of the current study was to determine the average age at initial diagnosis of BC between 2010 and 2022 and to assess any shifts in the proportions of early-onset cases of BC in patients treated by gynecologists and general practitioners in Germany.

## Methods

### Database

This study was based on data from the Disease Analyzer database (IQVIA), which contains basic demographic data, diagnoses, and drug prescriptions obtained directly and in anonymous format from computer systems used in office based practices of general practitioners and specialists [17]. The database covers approximately 3–6% of all outpatient practices in Germany. It has previously been shown that the panel of practices included in the Disease Analyzer database is representative of private practices in Germany [17]. Finally, this database has already been used in previous studies focusing on breast cancer [18–20].

### Study population

This retrospective cross-sectional study included adult female patients (≥ 18 years) with an initial breast cancer diagnosis (ICD-10: C50) in 2010 or 2022 in 300 general and 95 gynecological office based practices in Germany, which continuously delivered data in 2010 and 2022.

### Outcomes and statistical analyses

We analyzed the average age at first cancer diagnosis as well as the proportions of patients with a diagnosis of breast cancer in three age groups (18–49, 50–65, and > 65) in 2010 and 2022. Differences between the average age in 2010 versus 2022 were tested using Wilcoxon rank tests, and the proportions for the three age groups in 2010 versus 2022 using chi-squared tests. These analyses were conducted separately for patients in general and gynecological practices. A *p*-value of < 0.05 was considered statistically significant. Analyses were carried out using SAS version 9.4 (SAS Institute, Cary, USA).

## Results

### Characteristics of the study sample

Table 1 shows the patient samples available for analysis. A total of 2359 patients were initially diagnosed with breast cancer in 2010 compared to 3228 in 2022. The number of patients per practice treated by gynecologists was much higher than the number treated by general practitioners in each year (19.3 vs. 4.8 in 2010 and 16.2 vs. 5.7 in 2022).

**Table 1:** Female patients with first breast cancer diagnosis documentation by gynecologist or general practitioner in 2010 and 2022 in the Disease Analyzer database

Year	Total number of patients			Patients per practice	
Year	Total	GPs	Gynecologists	GPs	Gynecologists
2010	3,259	1,426	1,833	4.8	19.3
2022	3,238	1,697	1,541	5.7	16.2

### Evaluation of the mean age at first breast cancer diagnosis

**Fig. 1:**
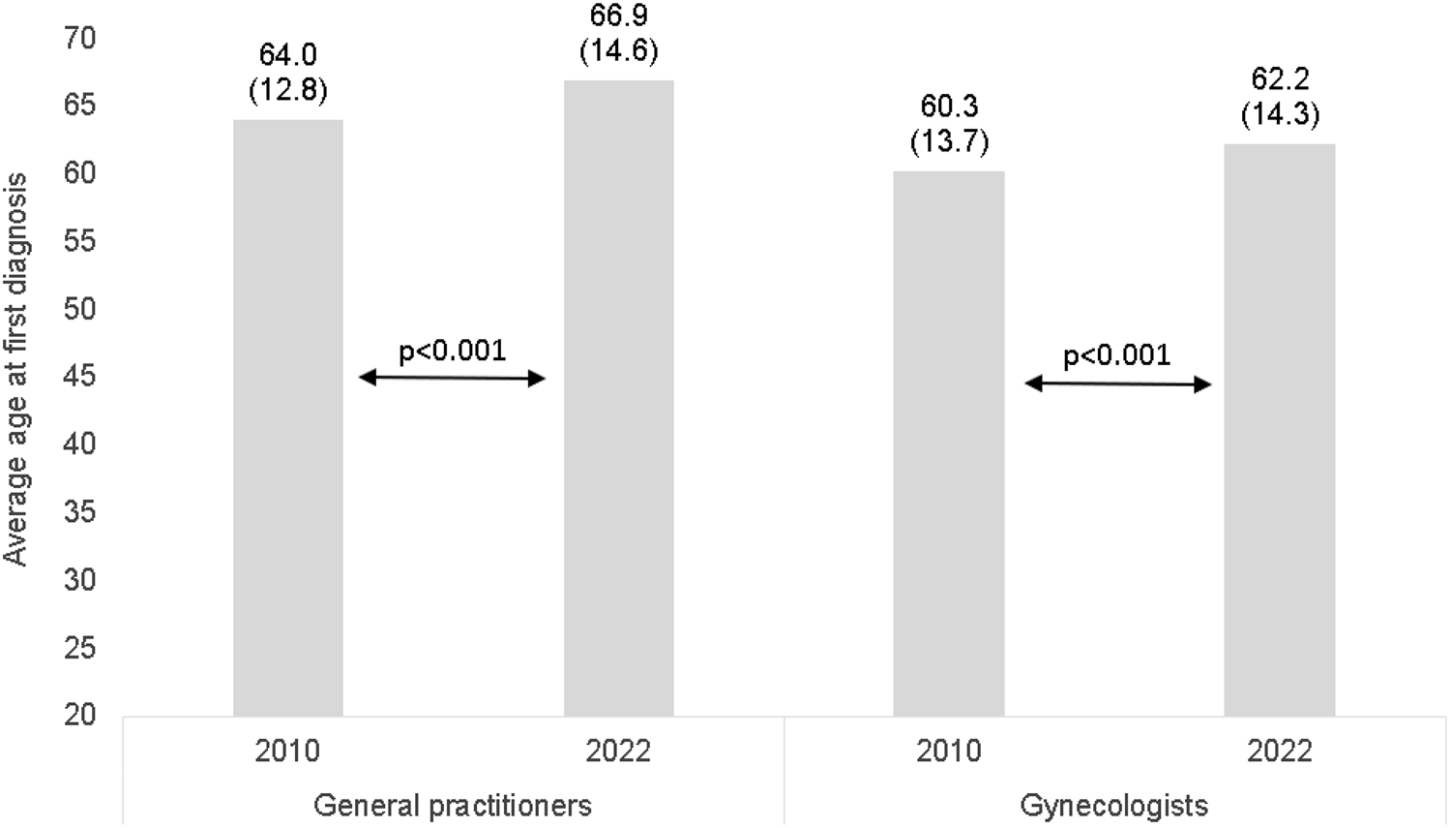
Average age at first documented breast cancer diagnosis between 2010 and 2022

Figure 1 shows the mean age at first documented breast cancer diagnosis in 2010 and 2022. The average age at the first diagnosis of breast cancer was significantly higher in 2022 than in 2010 in both GP practices (66.9, standard deviation (SD): 14.6 years versus 64.0; SD: 12.8 years, *p* < 0.001) and gynecological practices (62.2; SD: 14.3 years versus 60.3; SD: 13.7 years, *p* < 0.001).

### Proportions of patients with new breast cancer diagnoses by age group

**Fig. 2:**
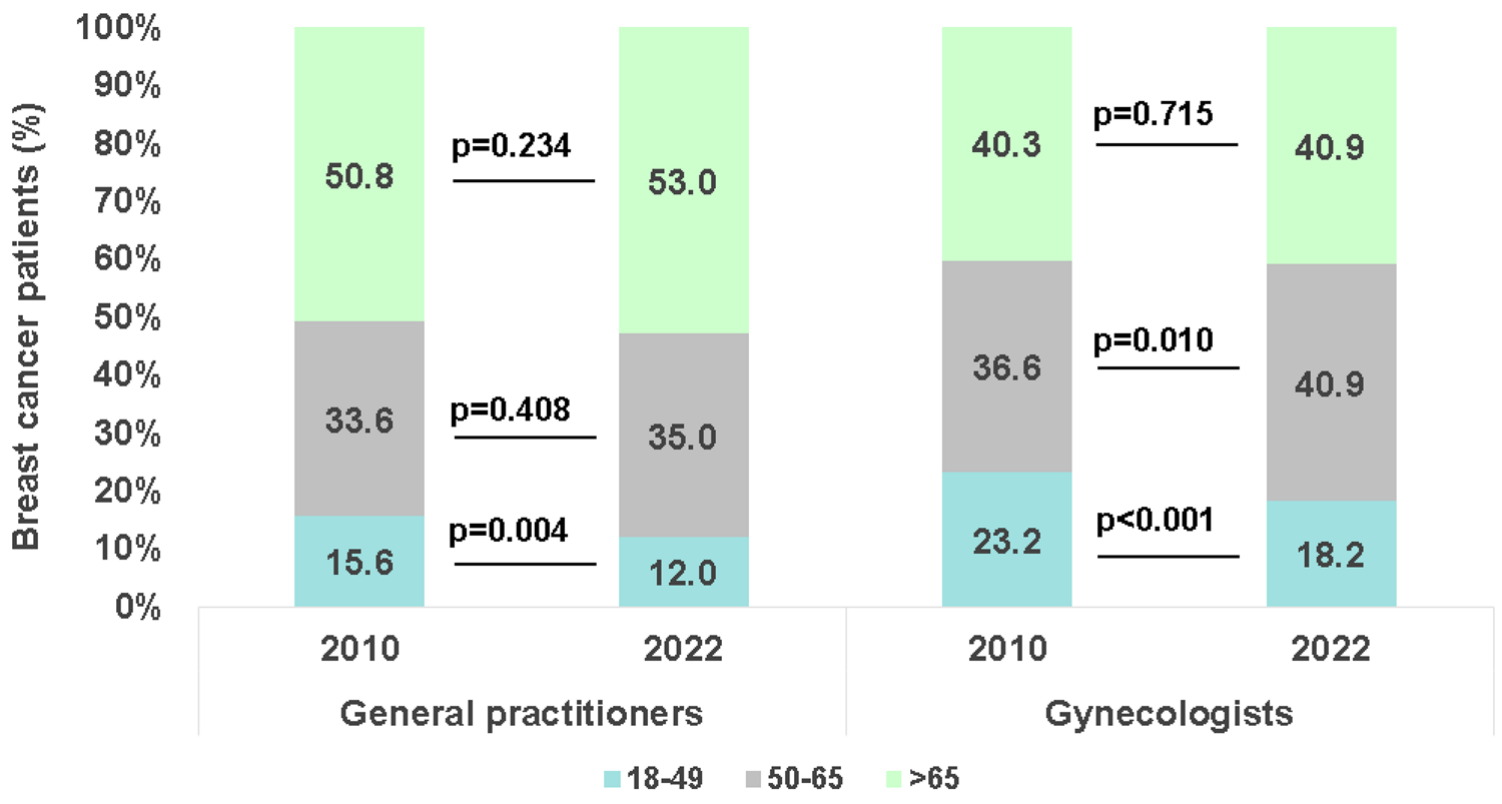
Age groups at first breast cancer diagnosis in 2010 and 2022

Figure 2 shows the proportions of patients with new breast cancer diagnoses in the three age groups. The proportion of early-onset cancers (defined as patient age < 50 years) decreased significantly from 15.6% in 2010 to 12.0% in 2022 (*p* = 0.004) in patients treated in GP practices and from 23.2% in 2010 to 18.2% (*p* < 0.001) in those treated in gynecological practices. Interestingly, we observed a significant increase in the proportion of new cancer diagnoses in the age group 50–65 years (36.6 to 40.9%, *p* = 0.010) in patients treated in gynecological practices but not in those treated in general practices. The proportion of patients aged > 65 did not change significantly from 2010 to 2022.

## Discussion

The results presented in this study indicate that the average age at first BC diagnosis has increased significantly in both general and gynecological practices between 2010 and 2022 (66.9 vs. 64.0 years and 62.2 vs. 60.3 years, *p* < 0.001, respectively). This observation can be interpreted in light of the effects of the German population-based mammography screening program (MSP), which was launched in 2005 and had been rolled out nationwide by 2009. In particular, women aged 50 to 69 are invited to attend biennial screenings in order to detect BC at an early stage and improve treatment outcomes [21]. Verdial and colleagues analyzed the National Cancer Institute’s Surveillance, Epidemiology, and End Results Program (SEER) data to examine breast cancer mortality, incidence, stage at diagnosis, and age at diagnosis with view to identifying associations between screening initiatives and breast cancer demographics. Among other results, the authors showed that the median age at first BC diagnosis had increased to 61 years over the study period (from 1973 to 2012) which was interpreted as a consequence of mammography screening [22, 23]. It is conceivable that MSP made it possible to detect and treat several types of BC precursor lesions (ductal and atypical lobular hyperplasia as well as ductal and lobular carcinoma in-situ) before they turned into invasive tumors, thereby increasing the age of patients at first BC diagnosis [24]. However, it should also be noted that worldwide age at BC diagnosis essentially reflected the median age of the general population. Bidoli et al. showed that from 2008–2012, each one-year increase in population age was associated with an increase in age at BC diagnosis of nearly half a year. Given the fact that the study period was some 12 years long (between 2010 and 2022), population ageing itself may also influence the mean age at first BC diagnosis [25]. In addition, changes within the last decade in modifiable risk factors such as obesity, smoking, alcohol consumption, physical activity and various other lifestyle factors may also have contributed to an increase in the age at which BC patients are diagnosed [26]. Besides this, the results of the Million Women Study (MWS) in 2003 suggested that an increase in BC cases under HRT had in turn affected prescription policy and contributed to a substantial decline in HRT prescriptions worldwide. Several studies have reported a decrease in BC rates along with a decline in HRT, highlighting a significant link between HRT and the occurrence of BC which may also be responsible for the increase in age at first BC diagnosis [27, 28].

Our study also revealed that the proportion of early-onset BC cases decreased over time, while the proportion of cases in the age group 50–65 increased in gynecological practices in Germany between 2010 to 2022. Due to their novelty and uniqueness to Germany, these data are of particular interest. In their review, di Martino and colleagues identified 12 publications from the US, eight publications from Europe, and a single study from South America that have shown increasing incidence trends of BC in younger women [13]. However, we observed the opposite trend for Germany, although the reasons for this phenomenon can only be guessed at since BC is a multifactorial, heterogeneous disease with a complex etiology. In line with our results, Katalinic et al. reported in a population-based analysis that breast cancer incidence is declining in women, including those under 50 years old. Based on data from a large quality assurance project, the authors concluded that the pattern of decline in breast cancer incidence in the German state of Schleswig–Holstein is consistent with the observed decrease in HRT utilization [16]. In addition, an inverse association between obesity and BC risk for premenopausal women has been reported in the past [29, 30]. Therefore, it is conceivable that the worsening obesity epidemic is responsible for the declining proportion of early-onset BC cases in Germany. The widespread and common use of the contraceptive pill, as well as better preventive management of hereditary breast carcinomas, may also explain the drop in the proportion of early-onset BC cases [31].

Finally, all our analyzed data from office based practices, should be compared with data from population-based cancer registries from Germany. Importantly, our study findings are consistent with cancer registry data from the Robert Koch Institute (RKI) which show a constant decrease in the proportion of the youngest age group (< 50 years) with breast cancer from 2010 to 2019 (2010: 13,066 BC cases, 17.8% to 11,005 BC cases, 15.4% in 2019) [32, 33]. Notably, our data cannot be directly compared to the RKI data, as we stratified by physician specialization, among other factors.

Comparing RKI cancer registry data from 2018 [32] to 2010 [33], the median age at BC diagnosis remained constant at 64 years. Our study found that the average age at first documented BC diagnosis in GP practices was 64 years, corresponding to the RKI data, while it was 52.2 years in gynecological practices, possibly due to mammography screenings and better diagnostics in gynecological practices. Moreover, we found that the average age at first BC diagnosis was higher in 2022 than 2010 in both GP and gynecological practices, but this differs from RKI data which show a constant median age at BC diagnosis of 64 years. However, it should be noted that comparing these data is limited by the lack of RKI cancer registry data for 2022.

In summary, further research is needed to investigate the underlying causes behind the changes observed in the incidence of early-onset BC between 2010 and 2022 in Germany.

### Strengths and limitations

Our retrospective cohort study has several strengths, including the use of the German Disease Analyzer (DA) large European outpatient database, which contains data from 250 gynecological and 1102 office based general practices in Germany, and has been validated with respect to the representativeness of the diagnoses it contains. The DA provides continuously updated data obtained directly from practice computers based on patient data such as diagnoses and demographic information and has been used successfully for numerous epidemiology studies. However, our study is also subject to several limitations, such as a lack of information on external confounding factors like alcohol and tobacco consumption and socioeconomic status. In addition, the DA lacks detailed information about co-diagnoses, molecular subtype of breast cancer, TNM classification, menopausal status, and other covariates such as hormone replacement therapy (HRT). Finally, as a retrospective database analysis, this study cannot establish causal relationships.

## Data Availability

Anonymized raw data are available on reasonable request.
